# Incidence of frailty among community-dwelling older adults: a nationally representative profile in China

**DOI:** 10.1186/s12877-019-1393-7

**Published:** 2019-12-30

**Authors:** Weihao Xu, Ya-Xi Li, Chenkai Wu

**Affiliations:** 1 Geriatric Cardiology Department of the Second Medical Center & National Clinical Research Center for Geriatric Diseases, Chinese PLA General Hospital, Beijing, China; 2 Global Health Research Center, Duke Kunshan University, Academic Building 3038, No. 8 Duke Avenue, Kunshan, 215316 Jiangsu China; 3 Duke Global Health Institute, Duke University, Durham, NC USA

**Keywords:** Frailty, Epidemiology, China, Health disparities

## Abstract

**Background:**

Frailty is a clinically recognizable state of reduced resilience to stressors and increased vulnerability to adverse outcomes. The majority of studies have focused on the prevalence and risk factors of frailty, while the incidence of frailty has not been well documented, especially in less developed regions including China—a country that has the largest aging population in the world. We investigated the incidence of frailty among non-frail Chinese older adults by sociodemographic characteristics, disease burden, and geographic region.

**Methods:**

Participants were 4939 adults aged ≥60 years from the China Health and Retirement Longitudinal Study, a cohort study of a nationally representative sample of middle-aged and older community-dwelling adults from 28 provinces in China. Frailty was assessed by an adapted version of the well-validated Fried’s physical frailty phenotype, in which five criteria were included: weakness, slowness, exhaustion, physical inactivity, and shrinking.

**Results:**

Over an average of 2.1 years of follow-up (10,514.2 person-years), the weighted incidence rate of frailty was 60.6 per 1000 person-years; the incidence rate was 28.8 and 86.6 per 1000 person-years for those who were initially robust and prefrail, respectively. Participants who were older and widowed, had lower education and household income, lived in rural areas, and had higher burden of chronic conditions had higher frailty incidence. Frailty incidence ranged from 44.8 per 1000 person-years in the Southeast to 93.0 per 1000 person-years in the Northwest.

**Conclusions:**

Incidence rate of frailty was 60.6 per 1000 person-years among community-living Chinese adults aged ≥ 60 years. Substantial sociodemographic and geographical disparities exist in frailty incidence.

## Background

Frailty is a clinical syndrome characterized by reduced reserve to stressors and increased vulnerability to adverse outcomes, resulting from age-related dysregulations across multiple physiological systems [1, 2]. Frailty is associated with higher risk of death, disability, and hospitalization, and higher healthcare expenditures, which would place a substantial burden on older persons, their caregivers, and health care resources [2–7]. Despite a burgeoning literature on the epidemiology of frailty, the majority of studies have focused on the prevalence and risk factors of frailty, while the incidence of frailty has not been well documented, especially in less developed regions [8].

Population aging is a worldwide phenomenon; over two-thirds of the world’s older population currently live in developing countries and the growth rate is accelerating [9]. China has the world’s largest aging population. In 2015, 201 million people were over the age of 60 years in China and this number is projected to more than double within 35 years, reaching 479 million in 2050. This enormous size of the older population in China has posed severe threats to its health care system [10, 11]. In prior work, we found that 7% of Chinese adults aged ≥60 years were frail and the prevalence of frailty varied substantially by sociodemographic and geographic region [12]. Knowing the incidence of frailty would be valuable in identifying Chinese elders who are at high risk for becoming frail and help depict a more complete picture of the epidemiology of frailty in China.

In the present study, we leveraged data from the China Health and Retirement Longitudinal Study (CHARLS) to identify the incidence of frailty among non-frail older adults in China by sociodemographic characteristics and geographic region.

## Methods

### Data and study participants

We used data from the CHARLS, a longitudinal cohort study comprising a nationally representative sample of middle-aged and older non-institutionalized adults from 28 out of 34 provincial-level administrative units in China. The baseline survey of the CHARLS was conducted between in 2011–2012. A total of 12,740 age-eligible households were contacted with a response rate of 80.5%. A total of 17,708 individuals residing in 10,257 households were recruited at baseline. Proxy interview was minimized by requiring the interviewer to request permission from the study headquarters; the national baseline rate of proxy interviews was 8.02% [13]. Follow-up survey is conducted biennially thereafter. The Ethical Review Committee at the Peking University approved the protocol of the CHARLS. Further details about the recruitment strategy, design, and sampling approaches of the CHARLS have been supplied elsewhere [13].

A total of 7681 participants were at least 60 years of age at baseline, of which 5301 had data on four or more frailty components. The survey module of physical activity was administered in a random sample of half of study participants and represented 53.0% of missing frailty component. Missing data for other four criteria were minimal: 2.4% for gait speed, 1.4% for handgrip strength, 0.7% for self-reported exhaustion, and 2.7% for weight loss. The present analysis includes 4939 persons who were ≥ 60 years and not frail at baseline (2011). The participants’ survival status and date of death (year and month) were collected during the follow-up visit in 2013; 191 (3.0%) and 296 (6.0%) died and were lost to follow-up, respectively.

### Frailty

Guided by the Fried’s physical frailty phenotype (PFP) framework, we used five criteria—slowness, weakness, exhaustion, physical inactivity, and shrinking—to assess frailty; this approach was previously created in the CHARLS to estimate the prevalence of frailty among Chinese older adults and has been validated for predictive validity [12]. Because the instruments were not identical, our operational definitions were modestly different from those previously described by Fried et al. in the Cardiovascular Health Study [2]. Participants met the slowness criterion if their walking speed (m/s), measured as the average of two timed walk tests over a 2.5-m course, was at or below the 20th percentile adjkusted for sex and standing height [12]. Participants met the weakness criterion if their grip strength, assessed by a Yuejian™ WL-1000 mechanical dynamometer, was at or below the 20th percentile adjusted for sex and body mass index (BMI) [12]. Grip strength for persons whose measuring position was unknown or lying down was coded missing. Participants met the exhaustion criterion if they answered “a moderate amount of time; 3 to 4 days” or “most of the time; 5 to 7 days” when asked “How often during the last week did you feel this way” to either of the two questions in the Center for Epidemiological Studies-Depression scale [14]: “I could not get going” and “I felt everything I did was an effort.” Participants met the inactivity criterion if they answered “No” to the question, “During a usual week, did you walk at least 10 minutes continuously?” Shrinking was defined as self-reporting loss of 5 or more kilograms in the previous year or having a BMI ≤18.5 kg/m^2^. Details of each of five criteria for defining frailty were provided in Table 1.

**Table 1:** Operational definition of five criteria for defining frailty among Chinese older adults

Criteria	Definition	
Slowness	Gait speed (Men):	Gait speed (Women):
	Height ≤ 163 cm: ≤0.45 m/s	Height ≤ 151 cm: ≤0.36 m/s
	Height > 163 cm: ≤0.48 m/s	Height > 151 cm: ≤0.43 m/s
Weakness	Grip strength (Men):	Grip strength (Women):
	BMI ≤20.6 kg/m^2^: ≤25.2 kg	BMI ≤20.0 kg/m^2^: ≤15.0 kg
	BMI 20.6–23.2 kg/m^2^: ≤28.5 kg	BMI 20.0–22.1 kg/m^2^: ≤17.5 kg
	BMI 23.2–25.9 kg/m^2^: ≤30.0 kg	BMI 22.1–24.8 kg/m^2^: ≤17.5 kg
	BMI > 25.9 kg/ m^2^: ≤30.0 kg	BMI > 24.8 kg/m^2^: ≤20.0 kg
Exhaustion	Felt “I could not get going” or “Everything I did was an effort” at least 3–4 days during the last week	
Inactivity	Did not walk at least 10 min continuously during a usual week	
Shrinking	Loss of 5 or more kilograms in the previous year or BMI ≤18.5 kg/m^2^	

*BMI* body mass index

We assessed the frailty level using the number of criteria met (0–5) at baseline and follow-up, respectively. Persons meeting none of the criteria were considered “robust”; those meeting 1–2 criteria were deemed “prefrail”; and those meeting at least 3 criteria were considered “frail”.

### Demographics, lifestyles, and diseases

Baseline demographic characteristics included age (60–64, 65–69, 70–74, 75–79, and 80+ years), sex, education (no formal education/illiterate, can read but did not finish elementary school, elementary school/traditional Chinese school, middle school, and high school or above), annual household income (categorized in quartiles: < 2160 Yuan, 2160–10,640 Yuan, 10,640–31,500 Yuan, and > 31,500 Yuan), marital status (married/living together, widowed, and others), current residence location (urban vs. rural), and geographical region (Northeast, North, Central, Southwest, South, Northeast, East, South Central, and Southeast). BMI was calculated as body weight (kilograms) divided by height (meters) squared. Participants reported whether they have been diagnosed with the following conditions: hypertension, diabetes, cancer (excluding minor skin cancers), cardiac disease (including myocardial infarction, coronary heart disease, angina, heart failure, or other heart problems), stroke, chronic lung diseases, liver disease, kidney disease, stomach or other digestive disease, and arthritis/rheumatism. We calculated the total number of chronic conditions and classified it as 0, 1, 2, and 3 or more.

### Statistical analysis

Participants were censored at the end of the follow-up period (November 2013) or at date of death, whichever came first. For participants who were lost to follow-up before the visit in 2013, they were censored at the middle date between their interview date at baseline and the end of the follow-up period. We estimated the incidence rates of frailty in the overall sample and by demographics including age, sex, education, marital status, and current residence location. We also identified the incidence of frailty by nine geographical regions. Incidence rates of frailty were compared for each socio-demographic variable using the Cox proportion hazard regression model. We conducted a multivariable-adjusted Cox model to identify the adjusted association of socio-demographic and disease with incidence of frailty.

Multistage probability sampling design of the CHARLS was appropriately accounted for by specifying the sampling weight, strata, and primary sampling unit parameters. All tests were two-sided with a significance level of *P* < 0.05. Multiple imputation with chained equations (MICE) was used to impute missing data for five binary indicators used to construct frailty, assuming that data were missing at random [15]. The MICE is a flexible missing data imputation technique that can appropriately deal with variables with non-normal distribution (e.g., binary) [16]. Each frailty criterion was modeled individually using logistic regression. Because participants with missing frailty indicators were more likely to have worse health status [17], we included a number of health measures (e.g., functional limitation and disability in activities of daily living) as auxiliary variables in the imputation models. Specifically, age, sex, marital status (married/living together, widowed, others), current residence location (urban and rural), education level (no formal education/illiterate, can read but did not finish elementary school, elementary school/ traditional Chinese school, middle school, and high school or above), standing height (continuous), body mass index, smoking status (current, previous, never), lower extremity functional limitation, upper extremity functional limitation, activities of daily living, instrumental activities of daily living, grip strength, gait speed, self-reported exhaustion level, self-reported physical activity level, and weight loss (all variables were measured at baseline) were used to impute missing data in five frailty criteria. For missing data in variables used for imputation, we created an indicator for categorical variables (i.e., missing-indicator method) and used mean imputation for continuous variables. Estimates were combined across 10 imputed datasets based on the Rubin’s rules [18]. All analyses were performed using Stata 15.0 (StataCorp, College Station, TX).

## Results

Compared with participants who had missing data in frailty measure (*n* = 1728) at the two-year follow-up, those with no missing data in the frailty measure (*n* = 3211) were younger, more likely to be male, married/living together, and rural residents, and had a higher prevalence of currently smoking, stomach disease and kidney disease, and a lower prevalence of hypertension and prefrailty at baseline (Table 2).

**Table 2:** Comparison in baseline characteristics between persons who with and without missing frailty data at follow-up

	No missing frailty data	Had missing frailty data	*p* ^*a*^
Characteristics	(*N* = 3211)	(*N* = 1728)	
Age, years, mean (SD)	67.0 (5.8)	68.2 (6.9)	<.001
Male, No. (%)	1691 (52.7%)	814 (47.1%)	.001
Education, No. (%)			<.001
No formal education or illiterate	1017 (31.7%)	693 (40.1%)	
Did not finish elementary school ^b^	708 (22.1%)	346 (20.0%)	
Elementary school ^c^	863 (26.9%)	399 (23.1%)	
Middle school	408 (12.7%)	180 (10.4%)	
High school or above ^d^	214 (6.7%)	111 (6.4%)	
Marital status, No. (%)			<.001
Married/living together	2664 (83.0%)	1356 (78.5%)	
Others ^e^	547 (17.0%)	371 (21.5%)	
Current residence, No. (%)			<.001
Urban	1085 (33.8%)	684 (39.6%)	
Rural	2126 (66.2%)	1045 (60.5%)	
Smoking			
Never	1813 (56.5%)	1003 (58.1)	.007
Previous	359 (11.2%)	235 (13.6)	
Current	1039 (32.4)	489 (28.3)	
Body mass index			.380
underweight	280 (8.7%)	150 (8.7%)	
normal	2087 (65.1%)	1090 (63.1%)	
overweight	838 (26.2%)	487 (28.2%)	
Hypertension, No. (%)	990 (30.9%)	610 (35.3%)	.005
Diabetes, No. (%)	228 (7.1%)	136 (7.9%)	.380
Cancer ^f^, No. (%)	32 (1.0%)	10 (0.6%)	.256
Cardiac disease, No. (%)	480 (15.4%)	290 (16.8%)	.145
Stroke, No. (%)	89 (2.8%)	59 (3.4%)	.246
Lung disease, No. (%)	466 (14.6%)	256 (14.8%)	.850
Liver disease, No. (%)	122 (3.8%)	67 (3.9%)	.968
Kidney disease, No. (%)	202 (6.3%)	74 (4.3%)	.010
Stomach disease, No. (%)	754 (23.5%)	352 (20.4%)	.022
Arthritis, No. (%)	1225 (38.2%)	651 (37.7%)	.750
ADL disability	535 (16.7%)	320 (18.4%)	.185
Frailty at baseline, No. (%)			<.001
Non-frail	1531 (47.7%)	708 (41.0%)	
Prefrail	1680 (52.3%)	1020 (59.0%)	

Abbreviations: *SD* standard deviation, *ADL* activities of daily living

^a^ *P*-values were obtained by a t test with continuous variables and a *χ*^2^ test for categorical variables

^b^ But capable of reading or writing

^c^ Including traditional Chinese school (i.e., Sishu)

^d^ Including graduate from high school, vocational school, college, or post-graduate

^e^ Including widowed, separated, divorced, and never married

^f^ Non-melanoma skin cancer was excluded

**Fig. 1:**
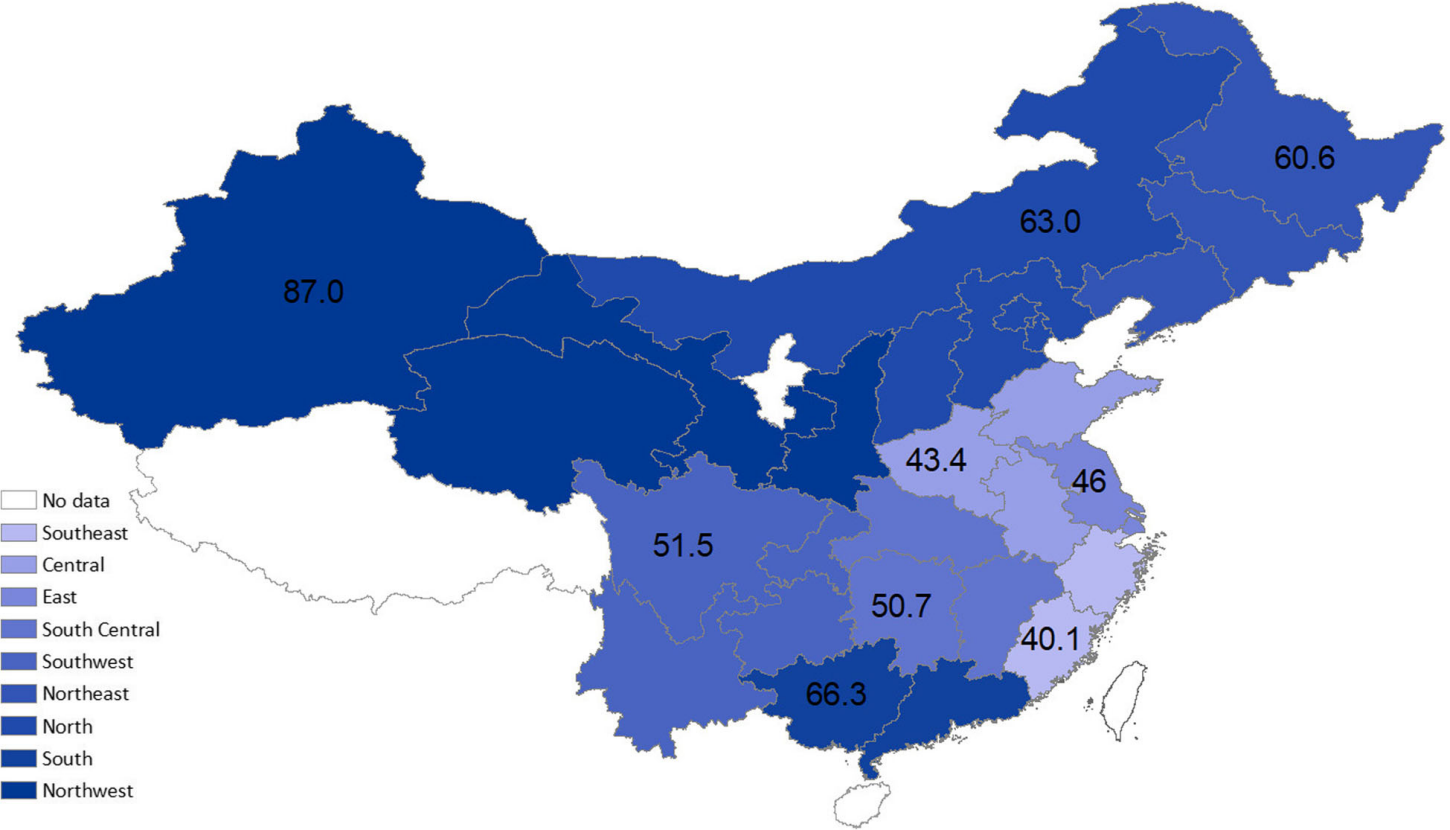
Age-adjusted incidence of frailty among participants who were not initially frail in 2011 by geographic regions, China Health and Retirement Longitudinal Study; weighted incidence rate (per 1000 person-years) was estimated at the weighted mean age in each region

Incidence rates of frailty are presented in Table 3. A total of 4939 older adults who were robust (45.1%) or prefrail (54.9%) at baseline were included. Over an average of 2.1 years of follow-up (10,514.2 person-years), the weighted incidence rate was 60.6 per 1000 person-years for the overall population; the incidence rate was 28.8 and 86.6 per 1000 person-years for those who were initially robust and prefrail, respectively (Table 3). Frailty incidence was significantly higher with advancing age, female sex, lower levels of education, lower annual household income, unmarried status, and rural residence; persons with different levels of chronic disease burden had different incidence of frailty. In multivariable-adjusted model, age, education, income, marital status (widowed vs. married), prefrail status, and disease burden (3+ chronic conditions vs. 0) persisted to be significantly associated with incidence of frailty; the difference between males and females largely attenuated and was no longer significant (Table 4). There was substantial geographic variation in frailty incidence in China (Fig. 1). The incidence estimates ranged over 2-fold from 44.8 per 1000 person-years in the Southeast to 93.0 per 1000 person-years in the Northwest. There was a clear trend of increasing frailty incidence from coastal areas to inland areas.

**Table 3:** Incidence of frailty among robust and prefrail persons in 2011, China Health and Retirement Longitudinal Study

	Demographic characteristics	Incidence of frailty per 1000 person-years		
	N (%)	Rate	95% CI	
Overall	4939 (100%)	60.6	54.2	68.0
Frailty at baseline***				
Robust	2227 (45.1%)	28.8	23.7	35.4
Prefrail	2712 (54.9%)	86.6	76.4	98.6
Age groups (years)***				
60–64	2025 (41.0%)	36.5	30.6	43.9
65–69	1297 (26.3%)	55.2	39.4	80.3
70–74	846 (17.2%)	65.6	52.7	82.9
75–79	522 (10.6%)	93.7	76.1	116.7
80–84	190 (3.9%)	152.2	118.8	198.4
85+	59 (1.2%)	219.6	154.0	329.9
Sex***				
Male	2542 (51.3%)	52.5	45.7	60.6
Female	2407 (48.7%)	69.1	58.6	82.1
Education***				
No formal education or illiterate	1673 (33.9%)	90.4	80.1	102.3
Did not finish elementary school ^a^	1057 (21.4%)	63.4	52.2	77.7
Elementary school ^b^	1255 (25.4%)	52.8	36.7	79.3
Middle school	600 (12.2%)	22.2	14.0	37.3
High school or above ^c^	352 (7.2%)	15.9	9.2	30.1
Annual household income***				
< 2160 Yuan	1163 (25.0%)	79.8	68.9	92.9
2160–10,640 Yuan	1163 (25.0%)	57.2	47.9	69.0
10,640–31,500 Yuan	1166 (25.1%)	40.4	32.7	50.7
> 31,500 Yuan	1159 (24.9%)	44.9	29.2	73.0
Marital status**				
Married	4000 (81.0%)	56.1	48.9	64.6
Widowed	844 (17.1%)	76.8	63.1	94.5
Others ^d^	95 (1.9%)	83.3	47.6	161.5
Current residence***				
Urban	1828 (37.0%)	53.7	42.2	69.6
Rural	3111 (63.0%)	65.9	59.4	73.2
Geographic region***				
Southeast China	328 (6.6%)	44.8	29.6	71.2
East China	243 (4.9%)	51.4	33.5	82.9
South Central China	698 (14.1%)	57.3	45.0	73.7
Southwest China	903 (18.3%)	57.4	46.7	71.4
Central China	1128 (22.8%)	65.9	59.4	73.2
Northeast China	332 (6.7%)	67.6	45.8	104.5
Northern China	545 (11.0%)	71.0	56.6	90.2
South China	446 (9.0%)	74.2	40.6	152.6
Northwest China	316 (6.4%)	93.0	70.8	124.9
Number of chronic conditions*				
0	1282 (26.0%)	46.7	38.3	57.6
1	1463 (29.7%)	46.9	38.7	57.3
2	1081 (22.0%)	46.7	37.8	58.4
3+	1099 (22.3%)	78.2	59.2	106.0

Abbreviations: *CI* confidence interval

^a^ But capable of reading or writing

^b^ Including traditional Chinese school (i.e., Sishu)

^c^ Including graduate from high school, vocational school, college, or post-graduate

^d^ Including separated, divorced, and never married

*** *P* < .001, ** *P* < .01, * *P* < .05 for comparison in incidence of frailty within each demographic variable

**Table 4:** Multivariable-adjusted association of socio-demographics and disease burden with incidence of frailty among robust and prefrail persons

	HR (95% CI)	*P*-value
Prefrail vs. robust at baseline	2.46 (1.86, 3.24)	<.001
Age (years)		
60–64	Ref.	
65–69	1.39 (1.01, 1.90)	.040
70–74	1.41 (1.02, 1.95)	.038
75–79	1.88 (1.35, 2.63)	<.001
80–84	3.11 (2.11, 4.59)	<.001
85+	3.55 (2.03, 6.22)	<.001
Female vs. male	1.03 (0.77, 1.37)	.849
Education		
No formal education or illiterate	Ref.	
Did not finish elementary school ^a^	0.76 (0.58, 0.99)	.048
Elementary school ^b^	0.72 (0.51, 1.01)	.059
Middle school	0.42 (0.25, 0.71)	.001
High school or above ^c^	0.23 (0.12, 0.44)	<.001
Annual household income		
< 2160 Yuan	Ref.	
2160–10,640 Yuan	0.74 (0.56, 0.98)	.038
10,640–31,500 Yuan	0.65 (0.49, 0.87)	.003
> 31,500 Yuan	0.81 (0.59, 1.12)	.204
Marital status		
Married	Ref.	
Widowed	1.39 (1.07, 1.81)	.014
Others ^d^	1.20 (0.51, 2.78)	.678
Rural vs. urban residence		
Number of chronic conditions		
0	Ref.	
1	1.09 (0.79, 1.50)	.601
2	1.14 (0.82, 1.58)	.426
3+	1.78 (1.28, 2.47)	.001

Abbreviations: *HR* hazard ratio; *CI* confidence interval

^a^ But capable of reading or writing

^b^ Including traditional Chinese school (i.e., Sishu)

^c^ Including graduate from high school, vocational school, college, or post-graduate

^d^ Including separated, divorced, and never married

## Discussion

In a nationally representative, prospective cohort study, we found that the incidence rate of frailty was 60.6 per 1000 person-years among community-living Chinese adults aged ≥ 60 years. Individuals who were prefrail at baseline versus robust were two times more likely to become frail. We found substantial socio-demographic disparities in frailty incidence among older adults in China. The incidence of frailty was higher with advancing age, lower education level, lower annual household income, widowed marital status, and higher burden of chronic conditions. These findings were consistent with a previous study that examined the incidence of frailty among older adults living in Beijing, China [19]. In addition, the disparities in frailty incidence among different socio-demographic subgroups we observed in the present study were consistent with our work and that of others examining the prevalence of frailty in China and other low- and middle-income countries [12, 20, 21]. Taken together, these results suggest that the subgroups with a high burden of frailty also have a high risk of becoming frail. More resources should be allocated to these high-risk populations to prevent and manage frailty.

We found that the crude incidence of frailty was significantly higher among females than males, which is in line with Zheng et al.’s study [19]. However, sex difference substantially attenuated and was no longer significant after adjusting for other socio-demographic factors. In an earlier study of older Mexican Americans, Espinoza et al. also found that the difference in frailty incidence was not significant between males and females [22]. In the present study, education and household income was associated with frailty incidence in both unadjusted and adjusted models. These results were consistent with prior studies showing that lower socio-economic status was a strong risk factor for frailty incidence in Western populations. Soler-Vila et al. found that a lower education and a manual occupation was associated with higher frailty risk among older women in Spain [23]. Using data from the Women’s Health Initiative, Woods and colleagues found that older women with a higher education and higher family income had lower incidence of frailty [24].

We found rural residents had higher incidence of frailty than those living in urban areas. These results were different from Zheng et al.’s study, in which urban participants were more likely to develop frailty than rural participants [19]. There are several potential explanations for these inconsistent findings. First, the age structure and urban-rural composition of the study population are different between the two studies. In addition, we examined the incidence rate (i.e., person-time) instead of incidence risk of frailty to appropriately handle persons who were lost to follow-up. Moreover, two different assessments of frailty—PFP and frailty index—were used.

In addition to rural-urban disparities, we found substantial geographic heterogeneity of frailty incidence, with inland areas having a much higher incidence than coastal areas. The incidence of frailty in Northwest was over 2-fold than that in the Southeast. These results were echoed by two recent studies showing regional disparities in the prevalence of frailty within the Chinese population [12, 25]. In a recent study, Chen and colleagues found that eastern provinces in China had much higher quality of healthcare—availability and accessibility to healthcare services—than the central and western regions [26]. Reducing huge regional health disparities is becoming one of the major challenges facing China, especially considering the slowdown of economic growth, enlargement of income gap, and acceleration in urbanization process. Taken together, these findings contribute to a better understanding of China’s increasingly growing regional disparities in access to health and health care resources [27] and may have implications for public health policy and practice.

Our study has many strengths. First, to our knowledge, we are among the first to examine the incidence of frailty in China, using a nationally representative sample of community-living older adults. Incidence of frailty has been reported in several western countries [8, 23], but research focusing on Chinese population, especially those using a nationally representative sample, has been limited. Second, we are among the first to identify geographic heterogeneity and rural-urban disparities in frailty incidence in China. We acknowledge several limitations. First, in the absence of a gold-standard diagnostic tool for frailty, our incidence estimates may not be comparable with those obtained from using alternative frailty assessments. The PFP has been validated in different study cohorts including the CHARLS [12, 28, 29], enabling it to be a desirable choice of frailty assessment. In addition, the five components of the PFP can be easily administered in clinical settings and are widely available in epidemiological studies. Second, we assessed frailty using a modified version of the original Fried’s PFP and modifications to the operational definitions of frailty criteria may lead to misclassification of frailty [30]. Third, our findings may not be generalizable to areas (e.g., Hainan) that are not part of the CHARLS and institutionalized elders. Forth, we did not account for competing risk of death when examining the incidence of frailty. In our study, only 3.0% of the study participants died during the follow-up period, which is unlikely to have a huge impact on the results.

## Conclusion

The incidence rate of frailty was 60.6 per 1000 person-years among community-living Chinese adults aged ≥ 60 years. We demonstrated higher rates of frailty among persons who were older, female, had lower education and lower income, and lived in rural areas. Substantial geographical also disparities exist in the incidence of frailty. Having a more complete picture of the epidemiology of frailty is the foundation for preventing frailty and reducing health disparities among Chinese older adults. More prevention efforts and resources should be allocated to reduce the frailty incidence among high risk population in China.

## Data Availability

All data used in the study can be accessed through: http://charls.pku.edu.cn/index/en.html after registering as a user.

## Abbreviations

BMI: Body mass index
CHARLS: China Health and Retirement Longitudinal Study
MICE: Multiple imputation with chained equations
PFP: Physical frailty phenotype

## References

[1] Fried L, Walston J, Hazzard WR, Blass JP, Ettinger WH, Jr Halter JB, Ouslander J (1998). Frailty and failure to thrive. Principles of Geriatric Medicine and Gerontology.

[2] Fried LP, Tangen CM, Walston J (2001). Frailty in older adults: evidence for a phenotype. J Gerontol A Biol Sci Med Sci.

[3] Armstrong JJ, Stolee P, Hirdes JP, Poss JW (2010). Examining three frailty conceptualizations in their ability to predict negative outcomes for home-care clients. Age Ageing.

[4] Hajek André, Bock Jens-Oliver, Saum Kai-Uwe, Matschinger Herbert, Brenner Hermann, Holleczek Bernd, Haefeli Walter E, Heider Dirk, König Hans-Helmut (2017). Frailty and healthcare costs—longitudinal results of a prospective cohort study. Age and Ageing.

[5] Ensrud KE, Ewing SK, Taylor BC (2007). Frailty and risk of falls, fracture, and mortality in older women: the study of osteoporotic fractures. J Gerontol Ser A Biol Med Sci.

[6] Romero-Ortuno R, Kenny RA (2012). The frailty index in Europeans: association with age and mortality. Age Ageing.

[7] Clegg A, Young J, Iliffe S, Rikkert MO, Rockwood K (2013). Frailty in elderly people. Lancet.

[8] Galluzzo L, O'Caoimh R, Rodriguez-Laso A (2018). Incidence of frailty: a systematic review of scientific literature from a public health perspective. Ann I Super Sanita.

[9] United Nations DoEaSA, Population Division (2017). World Population Prospects: The 2017 Revision, custom data acquired via website.

[10] Hu S, Tang S, Liu Y, Zhao Y, Escobar ML, de Ferranti D (2008). Reform of how health care is paid for in China: challenges and opportunities. Lancet.

[11] Tang S, Meng Q, Chen L, Bekedam H, Evans T, Whitehead M (2008). Tackling the challenges to health equity in China. Lancet.

[12] Wu Chenkai, Smit Ellen, Xue Qian-Li, Odden Michelle C (2017). Prevalence and Correlates of Frailty Among Community-Dwelling Chinese Older Adults: The China Health and Retirement Longitudinal Study. The Journals of Gerontology: Series A.

[13] Zhao Y., Hu Y., Smith J. P., Strauss J., Yang G. (2012). Cohort Profile: The China Health and Retirement Longitudinal Study (CHARLS). International Journal of Epidemiology.

[14] Radloff LS (1977). The CES-D scale a self-report depression scale for research in the general population. Appl Psychol Meas.

[15] White IR, Royston P, Wood AM (2011). Multiple imputation using chained equations: issues and guidance for practice. Stat Med.

[16] Azur MJ, Stuart EA, Frangakis C, Leaf PJ (2011). Multiple imputation by chained equations: what is it and how does it work?. Int J Methods Psychiatr Res.

[17] Theou O, Brothers TD, Mitnitski A, Rockwood K (2013). Operationalization of frailty using eight commonly used scales and comparison of their ability to predict all-cause mortality. J Am Geriatr Soc.

[18] Campion WM (1989). Multiple imputation for nonresponse in surveys - Rubin,Db. J Marketing Res.

[19] Zheng Z, Guan SC, Ding H (2016). Prevalence and incidence of frailty in community-dwelling older people: Beijing longitudinal study of aging II. J Am Geriatr Soc.

[20] Harttgen K, Kowal P, Strulik H, Chatterji S, Vollmer S (2013). Patterns of frailty in older adults: comparing results from higher and lower income countries using the survey of health, Ageing and retirement in Europe (SHARE) and the study on global AGEing and adult health (SAGE). PLoS One.

[21] Hoogendijk EO, Rijnhart JJ, Kowal P (2018). Socioeconomic inequalities in frailty among older adults in six low-and middle-income countries: results from the WHO study on global AGEing and adult health (SAGE). Maturitas.

[22] Espinoza SE, Jung I, Hazuda H (2010). Lower frailty incidence in older Mexican Americans than in older European Americans: the San Antonio longitudinal study of aging. J Am Geriatr Soc.

[23] Soler-Vila H, Garcia-Esquinas E, Leon-Munoz LM, Lopez-Garcia E, Banegas JR, Rodriguez-Artalejo F (2016). Contribution of health behaviours and clinical factors to socioeconomic differences in frailty among older adults. J Epidemiol Commun H.

[24] Woods NF, LaCroix AZ, Gray SL (2005). Frailty: emergence and consequences in women aged 65 and older in the Women's Health Initiative observational study. J Am Geriatr Soc.

[25] Ma LN, Tang Z, Zhang L, Sun F, Li Y, Chan P (2018). Prevalence of frailty and associated factors in the community-dwelling population of China. J Am Geriatr Soc.

[26] Chen S, Guo L, Wang Z (2019). Current situation and progress toward the 2030 health-related Sustainable Development Goals in China: A systematic analysis. PLoS Med.

[27] Wang H, Zhang L, Zou H-F (2012). Regional disparity in health and health care in China: China Economics and Management Academy, Central University of Finance and Economics.

[28] Bandeen-Roche K, Xue Q-L, Ferrucci L (2006). Phenotype of frailty: characterization in the women's health and aging studies. J Gerontol Ser A Biol Med Sci.

[29] Sutton JL, Gould RL, Daley S (2016). Psychometric properties of multicomponent tools designed to assess frailty in older adults: a systematic review. BMC Geriatr.

[30] Theou O, Cann L, Blodgett J, Wallace LM, Brothers TD, Rockwood K (2015). Modifications to the frailty phenotype criteria: systematic review of the current literature and investigation of 262 frailty phenotypes in the survey of health, Ageing, and retirement in Europe. Ageing Res Rev.

